# ‘Leanomics’ in healthcare: a three-year quality improvement study on the financial impact of a modified Kanban system in hospital storerooms

**DOI:** 10.1136/bmjoq-2025-003416

**Published:** 2025-11-12

**Authors:** Kenneth Jun Logrono, Belal Salem Mufadi Zu'bi, Raana Siddiqui

**Affiliations:** 1 Nursing Inpatient and Nursing Quality Improvement, Hamad General Hospital, Hamad Medical Corporation, Doha, Qatar; 2 Medical Nursing Inpatient, Hamad General Hospital, Hamad Medical Corporation, Doha, Qatar; 3 Hamad Healthcare Quality Institute, Hamad Medical Corporation, Doha, Qatar

**Keywords:** Lean management, Human factors, PDSA, Quality improvement, Statistical process control

## Abstract

**Background:**

Manual inventory management in hospital storerooms often relies on visual estimation, leading to inaccuracies and inefficiencies such as overstocking and out-stocking. Our audit revealed that a medical inpatient unit incurs weekly consumable costs of QAR 31 000 (US$8500), underscoring the financial impact of these inefficiences. While traditional Kanban systems have proven financially effective in specialty units, their use in inpatient settings is limited, and data on their financial impact in Middle Eastern and North African (MENA) healthcare systems are scarce. This study aims to redesign the traditional Kanban system and evaluate its long-term financial and operational impact.

**Methods:**

We applied the Model for Improvement framework while using Plan-Do-Study-Act cycles to test and refine interventions. The traditional Kanban system was redesigned by introducing replenishment triggers, adopting bin systems, implementing Kanban boards, and standardizing Kanban quantities based on the frequency of consumable use. Impact was assessed using statistical process control charts generated with QI Macros software. Outcome measures included total weekly consumable costs; process measures assessed staff compliance with the Kanban system; and balance measures tracked out-stocking rates and staff satisfaction.

**Results:**

Over three years, the modified Kanban system reduced weekly costs by 40–50%, from QAR 31 000 (US$8500) to QAR 19 000 (US$5100) during testing and stabilised at QAR 16 000 (US$4300) post-implementation. Staff satisfaction increased from 79% to 90%, driven by improved workflow and inventory tracking. Out-stocking rates declined from 0.04 to 0.02 per 1000 inpatient days during testing, ultimately reaching near zero after implementation. Compliance improved from 76% to 95%, directly contributing to both cost savings and operational efficiency.

**Conclusion:**

The modified Kanban system effectively reduces costs, enhances staff satisfaction and improves operational efficiency by minimising stockouts. This study underscores the value of quality improvement and lean methodologies, such as Kanban, in optimising healthcare supply chains and reducing waste.

**WHAT IS ALREADY KNOWN ON THIS TOPIC**

- Lean and Kanban methodologies have been shown to enhance efficiency, minimise waste and generate financial and economic benefits across diverse healthcare settings. Nonetheless, there remains a need for more structured frameworks that comprehensively quantify both the operational and financial impacts of these interventions.

**WHAT THIS STUDY ADDS**

- Building on existing evidence, this study highlights the importance of designing sustainable, data-driven processes that systematically and sustainably apply Lean principles to optimise financial outcomes, with a particular focus on hospital storerooms. Unlike previous reports, this presents a detailed framework for measuring and articulating the combined financial and operational benefits of Lean-driven interventions, thereby advancing the assessment of economic value in healthcare process improvement.

**HOW THIS STUDY MIGHT AFFECT RESEARCH, PRACTICE OR POLICY**

- This study provides researchers and improvement teams with a novel evaluation framework, enables healthcare organisations to assess cost-effectiveness and informs policies on Lean-based funding and application strategies.

## Introduction

### Problem

 Managing the healthcare supply chain has long been a complex and fragmented challenge that we have directly observed affecting the availability of consumables on the front lines. At our large referral hospital in Qatar, we relied on a demand-based ‘eyeballing system’, where nurses visually estimated supply needs. This often resulted in overstocking, understocking and frequent inaccuracies. Through our audits, we found that a single inpatient unit spent an average of QAR 31 000 (US$8500) per week. We also identified that 10–20% of consumables were discarded due to expiration, while stockouts often occurred 20–30 times per 1000 patient days.

### Rationale

Patient care relies on essential support activities such as inventory management, purchasing and supply distribution.^1^ Collectively referred to as healthcare logistics or supply chain management, these processes aim to ensure the timely delivery of the right products in the correct quantity and quality, thereby minimising stock-related issues.^1 2^ Since logistics can account for 30–40% of a hospital’s operating budget,^3^ they are frequently targeted for improvement.

### Available knowledge

Most unit storerooms in our hospital applied the 5S methodology to address supply issues such as stockouts, waste and rising costs. However, these efforts often failed to deliver sustainable financial or operational benefits, as 5S is frequently implemented as a standalone solution rather than as part of a broader Lean strategy.^4 5^

Our team drew inspiration from the Toyota Production System (TPS), particularly its just-in-time (JIT) principle.^6^ A key JIT tool is Kanban,^2^ which uses visual cues and a pull system to align inventory with demand and reduce waste.^1 2^

Studies have shown that Kanban reduces costs in hospital storerooms by enhancing waste control and improving frontline responsiveness.^1 2 7^ However, it is typically digitised and confined to specialty areas such as operating rooms (ORs) and emergency departments (EDs), where storerooms are usually managed by a single staff member.^2 8^ Its application in general inpatient units remains uncommon due to service complexity and high inventory volumes. Literature that clearly structures the assessment of Lean implementation in the healthcare supply chain remains scarce.^7^ Furthermore, data on the sustainable financial impact of Kanban in Middle Eastern and North African (MENA) healthcare settings are also limited.^1^ Therefore, in this study, we aimed to (1) evaluate its long-term financial effects in inpatient storerooms and (2) describe the modified Kanban system implemented by our team.

## Methods

### Context

This study was conducted at Hamad General Hospital (HGH), a 600-bed tertiary teaching facility. The focus was the Medical Inpatient Department—one of the hospital’s largest—comprising 15 units: three acute, five specialty and seven general units, each with a 20–30 bed capacity. The department manages both acute and long-term care for patients with complex conditions. The pilot was implemented in one acute unit (in 6 North 3) serving both acute and ambulatory cases.

Consumables in HGH are supplied through a centralised system serving all hospitals in the network. Unit managers order supplies electronically via i-Procurement, which facilitates requests and approvals from the central store. Most medical consumables are delivered two to three times weekly, while intravenous fluids (IVFs), sourced from the pharmacy, are delivered separately every Tuesday by a designated store man.

### Model for Improvement (MFI)

This study was guided by the Model for Improvement (MFI) from the Institute of Healthcare Improvement (IHI),^9^ a widely used quality improvement (QI) framework that accelerates meaningful change in healthcare systems through structured testing and measurement. It centres on three key questions:

1. What are we trying to accomplish?
2. How will we know a change is an improvement?
3. What changes can we make that will lead to improvement?

These questions shaped the design, implementation and evaluation of our interventions to address supply inefficiencies and enhance cost-effectiveness. While using iterative Plan-Do-Study-Act (PDSA) cycles, we tested and refined the modified Kanban system in real settings, ensuring the changes were evidence-based, measurable and aligned with our operational and financial goals.

#### Aim: What are we trying to accomplish?

We aim to reduce the weekly total cost of consumable supplies in the medical inpatient units by 50%, from an average of QAR 31 000 (US$8500) to QAR 15 000 (US$4250), by March 2022 (over the end of 12 months), through the implementation of a modified Kanban system.

#### Measures: How will we know that a change is an improvement?

##### Outcome Measure: Weekly Total Cost of Consumable Supplies

The outcome measure used by our team is the weekly total cost of all consumable supplies in the pilot unit. This includes both direct supply items (e.g., syringes, dressings and IV lines) and indirect nursing-related supply costs (e.g., items ordered during emergency restocking or for administrative purposes). Cost data are captured through the automated i-Procurement system, which logs each issued item, its unit price and delivery frequency. By aggregating the cost of all consumable items delivered to the unit each week, we calculate the total weekly expenditure.

This measure reflects the financial impact of supply chain performance and helps monitor the effect of interventions—such as transitioning to cost-efficient alternatives or improving inventory flow through the redesigned Kanban system. Regular data-over-time analysis allows the team to identify special and common causes such as inefficiencies and opportunities for sustained cost optimisation.

##### Process measure: Percentage of Staff Compliance

Staff compliance is measured by tracking adherence to the new system. Every Kanban card not placed on the board as required is considered a defect or instance of non-compliance. This measure ensures that staff follow the standardised process, which is essential for maintaining efficient inventory flow, preventing unnecessary out-stocking and improving overall supply chain reliability.

##### Balance measure: Out-stocking Rate and Staff Satisfaction

The out-stocking rate tracks how often consumable items reach zero stock levels. This is a critical measure, particularly for emergency and high-priority items, as it ensures that predefined stock quantities and Kanban levels are sufficient to meet patient care demands. By monitoring stockouts, our team can adjust inventory levels, refine demand forecasts and prevent disruptions in care delivery. Staff satisfaction was also tracked as a balance measure, reflecting the impact of the modified Kanban system on staff experience.

Our team ensures the completeness and accuracy of data by cross-checking records from the automated system with manual inventory logs and conducting regular audits. Additionally, data validation processes are built into the system to flag discrepancies. Regular training sessions for our improvement team help maintain accurate data entry and reduce human error. Monitoring the consistency and integrity of this data-over-time allows us to verify the reliability of our outcome and process measures.

### Change Ideas and Interventions: What changes can we make that will result in improvement?

We redesigned the original Kanban system iteratively, drawing from best practices in literature, from our clinical experience and based on our existing workflows. We developed a Driver Diagram to identify the key factors necessary to achieve our aim and to visually present our theory of change. The primary drivers include senior leadership and sponsor engagement, education and awareness sessions for leaders and frontline staff, the redesigning of storeroom processes through iterative testing and implementation of the modified Kanban system and a focus on staff experience and satisfaction. Each primary driver is supported by secondary drivers and targeted change ideas to facilitate effective execution of our change strategy. The Driver Diagram is treated as a dynamic tool and is regularly updated based on our learnings from each PDSA cycle.

**Figure 1:**
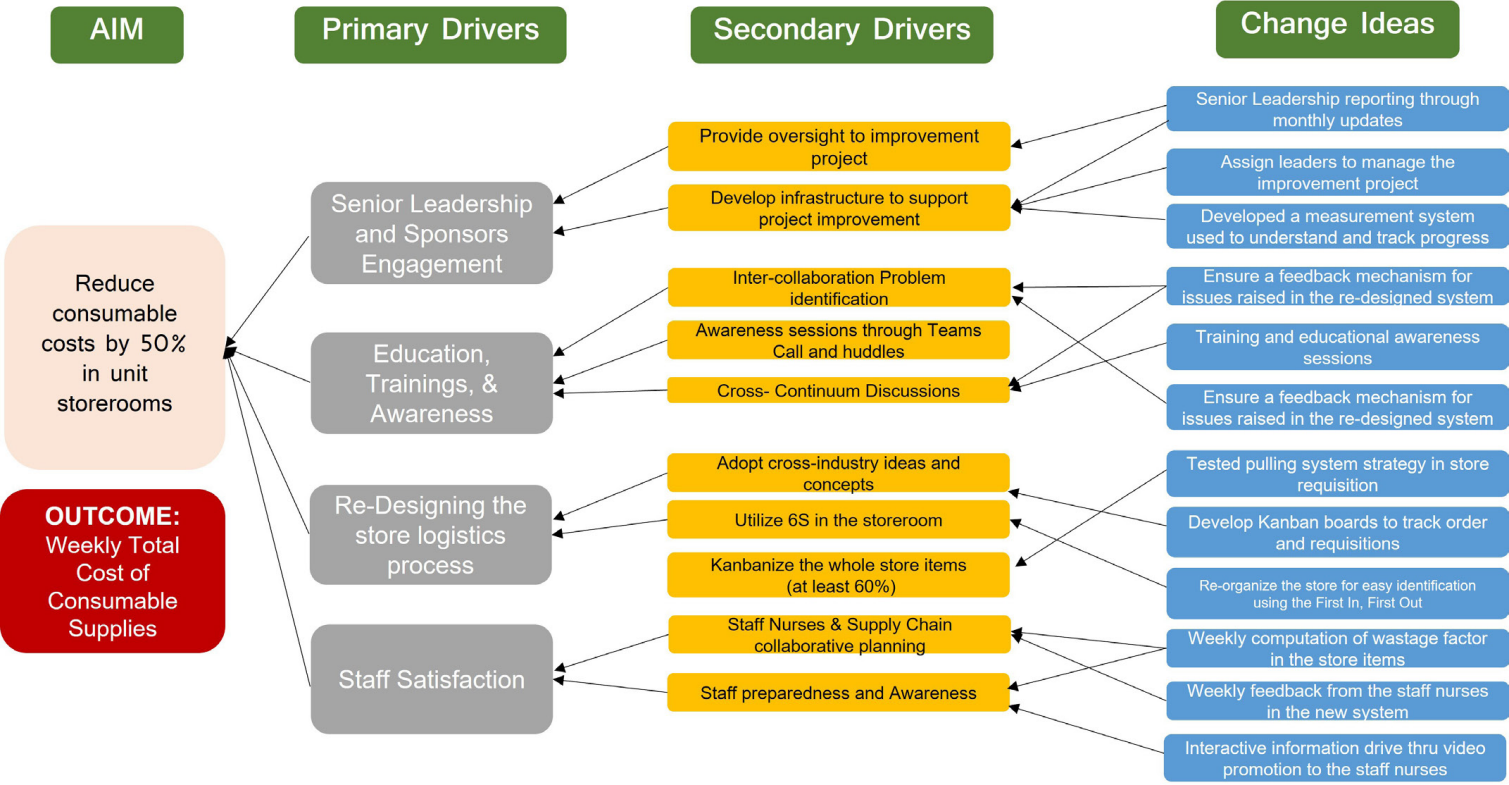
Driver diagram outlining the project aim, primary and secondary drivers and associated change ideas used to guide the implementation of the modified Kanban system in medical inpatient units.

During the initial testing, we encountered challenges with the original Kanban system, particularly its reliability as it solely used bins for signals. The problem of using bins was that it took up too much space,^3 8^ which limits the type of consumables being contained inside each unit storeroom. To address this, we formed an improvement team to develop a leaner, more efficient system tailored to our storeroom’s workflow and layout. The team consists of mostly staff nurses, a unit head nurse, a director of nursing and an improvement specialist and coach. The system was redesigned into three phases:

### Phase 1: Flow analysis of the consumable items

In this phase, we studied the movement of consumables based on our pilot unit’s scope of service, bed capacity and utilisation rate of the consumables. We quantified weekly consumption and categorised items into: Fast-moving items—these items were at high risk of stockouts and frequently used supplies, such as IVFs, IV cannulas, kidney basins, tourniquets, plasters, etc; and slow-moving items—less frequently used supplies that often reached expiration and were at a higher risk of becoming a waste.

Our flow analysis of consumables involves mapping the supply chain by documenting the flow of items from suppliers to storage and staff use. It includes inventory tracking,^8^ where stock levels, order quantities and replenishment rates are analysed to ensure optimal inventory. Lead time analysis,^7^ assesses the time from order placement to delivery and restocking, helping identify delays and inefficiencies. Finally, demand forecasting^10^ analyses historical usage patterns to predict future demand, ensuring the right quantities of consumables are available when needed.

### Phase 2: Kanbanisation and stock control

Stock levels were set based on historical usage to balance availability and overstocking. For each item, total stock equalled average weekly consumption plus a safety buffer—the Kanban level. This level was typically 20% of total stock but adjusted based on usage and resupply lead time. Kanban cards were placed at the reorder point, matching the quantity needed during the resupply window or lead time. For example, with a weekly demand of 50 units of normal saline bags and 2–3 days lead time (~10 units/day), stock was set at 60 units, with the Kanban card at 20 units—not 12—to prevent stockouts. Kanban levels were regularly reviewed and adjusted based on demand or delivery changes. This approach improved stock visibility, reduced emergency restocking and ensured uninterrupted clinical operations.

Based on the usage patterns and to accommodate items of different sizes, we redesigned the original kanban system to three sub-systems (see online supplemental figure 1):

1. *Divider system*: We used plastic dividers to separate the total stock from the Kanban level, attaching a Kanban card as a reorder signal or a replenishment trigger.
2. *Bin system*: For fast-moving items such as IVFs, we implemented a bin-based system, using three to four bins with Kanban cards to indicate when replenishment was needed.
3. *Rubber band system*: For slender, coiled items like nasogastric tubes (NGTs) and suction tubes, we applied rubber bands to mark the Kanban level, ensuring efficient storage and easy tracking of these kinds of items.

### Phase 3: Create a Kanban board and 6S implementation

After implementing Kanban for the consumables and assigning them to the appropriate subsystems, our team introduced a Kanban board^6^ with three sections to streamline the ordering process and minimise handoffs among staff:

- *To Order*: When stock reaches the Kanban level, we place a Kanban card here to signal the need for replenishment.
- *Ordered*: Cards move to this section once we place an order.
- *Supplied*: After delivery (typically within 2–3 days), we move the cards to this section, indicating that items should have been restocked.

To sustain the redesigned system, we also applied the 6S methodology—Sort, Set in Order, Shine, Standardise, Sustain, (and Safety)—ensuring continuous improvement and efficiency in our storerooms.

Following the redesigned Kanban system, we tested changes iteratively through sequential PDSA cycles, gradually increasing our confidence in the effectiveness of our change ideas. Each cycle involved four phases: Plan (establish objectives), Do (implement the plan), Study (evaluate the results) and Act (make necessary adjustments).^10^ The testing phase of this study lasted for 14 weeks, followed by the implementation phase, which also included spreading and scaling to other units and services.

**PDSA #1: Robust staff education and awareness of the modified Kanban system**

In the Plan phase, we aimed to educate all pilot unit staff using the teach-back method. During the Do phase, a three-week session was held for nurses, store men and aides, covering Kanban’s process, benefits and goals. In the Study phase, 70–80% showed correct understanding, though 20% of aides missed the training. In the Act phase, we planned follow-up education and moved forward with system testing.

**PDSA #2: Small scale testing of the modified Kanban system**

In the Plan phase, we initiated small-scale testing of the modified Kanban system using a frequently used item—tourniquets—over three weeks. During the Do phase, we monitored compliance, which ranged from 40–70%, with no stock-outs observed. In the Study phase, we identified the need for improved staff adherence. In the Act phase, we introduced reminder education via huddles and infographics and expanded testing to five fast-moving items over the next four weeks.

**PDSA #3: Scale-up the testing**

In the Plan phase, we assessed the Kanban system’s effectiveness for fast-moving items and the impact of infographics and huddle reminders. During the Do phase, we expanded testing to five high-use items over 4 weeks, with additional support from spot-check demonstrations. In the Study phase, compliance improved to 70–80%, but IVFs and syringes experienced stock-outs. We addressed this by adjusting stock levels and introducing a bin system. In the Act phase, we focused on refining the bin system for IVFs and applied similar adjustments to other items.

**PDSA #4: Scale-up the testing**

In the Plan phase, we aimed to evaluate the bin system for IVFs and syringes by monitoring compliance and stock-outs over four weeks. During the Do phase, we tracked and reported these metrics weekly using a visual board and developed standard compliance guidelines. In the Study phase, no IVF stock-outs occurred, overall stock-outs remained low (0.02 per 1000 patient days) and compliance rose to 80–90%. In the Act phase, we adopted the redesigned Kanban system for 60–70% of consumables, setting exclusion criteria for the rest.

**PDSA #5: Full implementation of the modified Kanban system**

After completing the previous cycles, we Kanbanised most consumables within one week. Team members acted as ‘secret shoppers’, monitoring compliance, stock-outs and costs. After one month, minor adjustments were made to Kanban levels and total quantities. Staff compliance remained at 80–90%. We plan to continue monitoring for 6–12 months to sustain improvements.

### Analysis

To evaluate improvements, we used statistical process control (SPC) charts to detect special and common cause variations.^11^ Data were entered into QI Macros, which auto-generated SPC charts. Control charts help visualise process performance over time, including in real time.^11^ We also assessed the process capability of the modified Kanban system to determine its financial sustainability. To ensure rigour and consistency, we followed the revised Standards for Quality Improvement Reporting Excellence (SQUIRE) 2.0 guidelines,^12^ which provided a structured framework for reporting our QI design, implementation and outcomes.

## Results

**Figure 2:**
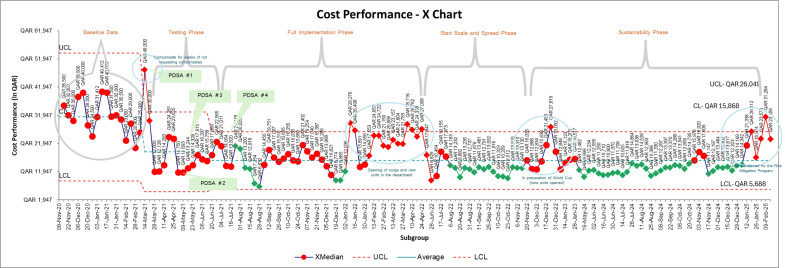
Weekly total cost of consumable supplies x-chart; (LCL, Lower Control Limit; UCL, Upper Control Limit)

The intervention led to a significant and sustained improvement in the management of supply costs, as demonstrated by the SPC chart. The primary outcome measure was the weekly cost of consumables from the hospital’s i-Procurement system. At baseline (18 weeks), the average weekly cost was QAR 31 000 (~US$8500). After implementing the modified Kanban system (14-week testing phase), costs dropped by 39% to QAR 19 000 (US$5130), with control limits from QAR 5000 to QAR 33 000 (US$1350–US$8910). This drop reflected early impacts of the intervention and continuous PDSA refinement.

Control limits were recalculated at key phases—during testing and full implementation—to reflect process stability. Continued PDSA cycles and improved staff compliance helped sustain changes.

After three years, weekly costs stabilised at QAR 16 000 (US$4320) with updated control limits (QAR 6000–QAR 26 000 or US$1620–US$7020).

Several special cause variations were observed using the IHI control chart rules. Intentional special causes, marked by green diamonds in the SPC charts, included a downward trend (Rule 3) from August 1o to August 29, 2021, and a cluster of two out of three points near a control limit (Rule 5) between December 19, 2022, and January 2, 2023. In addition, Rule 2, which signifies a shift, was triggered when eight or more consecutive points fell on one side of the average line. This rule signals a significant and sustained change in the process. We observed three such shifts during the sustainability phase of the project (from August 6 to November 6, 2022; from January 29, 2023 to October 20, 2024; and again from November 17 to December 20, 2024). These extended runs of data points below the centre line suggested sustainable reduction of costs following full system implementation.

However, unintentional variations were noted during unit expansions and service launches, such as surge units (January to June 2022), FIFA World Cup preparation (late 2022) and a unit’s shift to long-term care service (December 2024 to January 2025). Despite fluctuations, most data points stayed within control limits, demonstrating the modified Kanban system’s long-term cost reduction and sustainability (online supplemental figure 2).

As the modified Kanban system introduced a new process, we created clear compliance guidelines for staff, including nurses, aides, clerks and housekeepers (online supplemental figure 2). Compliance was measured by direct observation: the number of staff following the system divided by total observations (minimum of 10), grouped weekly. During the 14-week testing phase, compliance averaged 76% (LCL: 47%–UCL: 100%), with initial low rates expected due to staff adjustment.

After education through infographics, video demos and leadership-led return demonstrations, compliance rose to 95% (LCL: 80%–UCL: 100%). The p-chart showed a process shift starting June 20, 2021, with data indicated a stable process with common cause variation. Improved compliance associated with cost reductions observed from September 19, 2021, confirming its direct impact on financial efficiency and highlighting the importance of consistent compliance monitoring.

**Figure 3:**
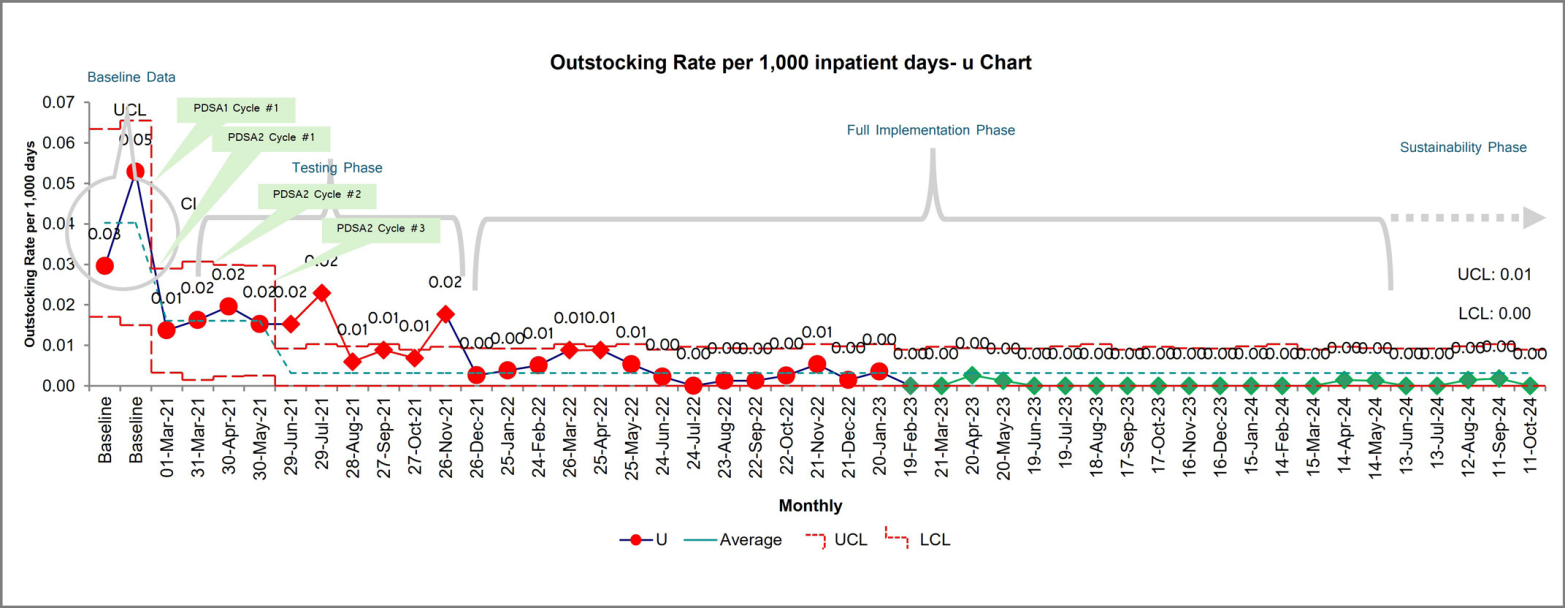
Out-stocking rate u-chart; (LCL, Lower Control Limit; UCL, Upper Control Limit)

While the implementation of the modified Kanban system effectively reduced supply costs during the testing phase, careful attention was given to avoid unintended consequences such as stock shortages. Standardising quantities and calculating Kanban levels are powerful cost-cutting strategies but can risk increased out-stocking.

Out-stocking was a key balance measure assessed through a u-chart, tracking the frequency of stock out incidents per 1000 inpatient days. The baseline out-stocking rate of 0.04 (20–30 stockouts) improved to 0.02 (11–15 stockouts) during the testing phase, coinciding with the period of significant cost reduction. This improvement was driven by PDSA #3, which successfully tested and refined the bin system and adjusted Kanban quantity levels for fast-moving items, demonstrating that cost efficiency was achieved without compromising supply availability.

Staff compliance significantly affected out-stocking rates. During testing, despite achieving 75% compliance, the stocking process showed instability attributable to a special cause variation, out-stocking rate of 0.02 (11–15 stockouts). This led to adjustments in quantities, Kanban levels and re-classifying item categories. Following these refinements, the system reduced out-stocking to 1–2 incidents, with a stable rate of 0 per 1000 patient days—indicating a controlled process tied to specific interventions.

Lower out-stocking rates improved staff satisfaction. Surveys showed staff often borrowed from other units, reducing patient care time. The Kanban system addressed this by streamlining inventory. During testing, satisfaction averaged 79% (LCL: 40%–UCL: 100%) (online supplemental figure 3). However, testing a bin system (PDSA #3) for high-use items effectively reduced stockouts, boosting staff satisfaction to 90–100% from April 18, 2021. The p-chart confirmed process stability and showed improved staff experience.

Overall, the modified Kanban system reduced costs, minimised stockouts and improved staff satisfaction. Lessons from data-driven variation analysis helped refine the system, ensuring sustained impact. As a result, it was scaled in late 2021 to 16 units, including paediatrics, surgery, neuroscience and emergency care, and, by early 2025, expanded to multiple hospitals across our health systems.

## Discussion

### Summary

While Lean and Kanban methodologies have been widely applied in healthcare to enhance efficiency and reduce waste, few studies have conducted rigorous economic evaluations—particularly within general inpatient settings in the MENA region. This study addresses that gap by presenting a data-driven financial assessment of a modified Kanban system implemented in inpatient unit storerooms. Unlike previous reports that emphasise process improvements or digitised Kanban in high-specialty areas (e.g., ORs and EDs), our innovation demonstrates how a low-tech, frontline-driven adaptation of Lean tools can yield measurable, sustained cost savings. This adds to the growing body of literature by offering context-specific insights into the financial value of Lean implementation in resource-constrained healthcare environments.

Over three years, the intervention led to a 40–50% cost reduction, a 50% decrease in stockouts and 80-90% staff satisfaction. These outcomes align with our aims to reduce inefficiencies while improving cost control and staff experience. Through iterative PDSA cycles and adoption of Lean principles, the system was continuously refined—reducing waste, limiting hand-offs, saving indirect nursing hours and improving expired-item tracking. These changes supported long-term sustainability and readiness for broader implementation.

Building on prior financial evaluations of Lean, this study also introduces the concept of *‘leanomics*’—a practical framework combining Lean and economic decision-making. This emerged during implementation when selecting lower-cost, functionally equivalent consumable items without sacrificing quality. Leanomics emphasises how operational decisions can drive both efficiency and financial value, offering a new lens for value-based improvement.

A key strength of the modified Kanban system is its potential scalability. Designed for facilities without digitised supply systems, its low-tech, adaptable format can be applied across similar hospital units and resource-limited settings. While broader adoption may require contextual adaptation, this approach offers a practical, cost-saving solution for improving supply chain performance in environments with comparable infrastructure.

### Interpretation

Few studies have explored Lean practices in healthcare supply chains, particularly their economic and workforce impacts.^7^ Our study contributes to the growing body of knowledge by demonstrating that a modified Kanban system achieved sustained 40–50% cost reductions—equivalent to savings of QAR 15 000 (~US$ 4000) weekly, or an estimated QAR 1.5 million (~US$ 420 000) annually in Kanbanised units. These results suggest strong potential for system-wide scalability.

This finding aligns with other Lean studies reporting up to 43% material cost reductions and 19% savings in consumables per patient.^1 7 12^ The limited success of standalone 5S efforts—often influenced by publication bias and weak integration^4^ —was addressed in our study through phased Kanban implementation combined with other Lean tools, such as visual management and 6S.

A key factor in our success was detailed flow analysis, which incorporated item usage, stockouts, bed capacity and service scope to optimise Kanban levels (typically ~20%) and standardise ordering processes.^7^ Initial staff compliance averaged 76%, with lower adherence attributed to unfamiliarity and unclear perceived benefits. However, through education, leadership support and the use of visual tools, compliance improved to 80–90%, consistent with studies identifying knowledge gaps as a common barrier.^12^

Tracking critical items (e.g., intubation tubes) highlighted the importance of expert-led flow analysis in establishing accurate reorder points.^1 2^ Occasional stockouts underscored the need for stronger macro-level supply coordination. Staff satisfaction improved in parallel, reflecting literature that shows Kanban enhances material availability and reduces non-nursing care tasks.^1 12^ Our low-tech adaptations—such as rubber bands and dividers—further improved organisation and minimised missing items, reinforcing findings from previous logistics improvement studies.^1 8^

Higher compliance reduced stockouts and increased staff satisfaction, creating a reinforcing cycle that emphasised the role of standardisation in complex workflows. Given the scarcity of Kanban research in MENA health systems,^1 7 12^ our findings provide additional evidence of its sustainability in operational, financial and workforce impacts. Finally, the integration of QI method and tools (e.g., MFI, PDSA cycles and SPC charts) and lean thinking supported timely supply delivery and improved patient care. However, long-term success depends on both systems redesign and the maturity of lean culture to sustain improvements.

### Limitations

Findings may have limited generalisability, especially to settings with different infrastructure, staffing or resources. The MENA context may not reflect other regions. Confounders like staffing changes, supply disruptions, bed capacity and shifting service scopes could have influenced results. Biases may exist in subjective staff satisfaction data and inaccuracy in tracking indirect nursing hours or exact cost savings.

To reduce confounding, we controlled staffing variables and used multiple data sources. PDSA cycles allowed real-time system refinement, and consistent measurement methods helped minimise bias and imprecision.

## Conclusions

In conclusion, our study shows that the modified Kanban system is an effective and sustainable innovation for healthcare supply chains, achieving sustainable cost savings, improved staff experience and streamlined workflows. Implemented in one department unit, it saved QAR 1.5 million (US$420 000) annually, demonstrating strong scalability with potential hospital-wide impact.

This study also introduces ‘*leanomics*’, linking lean principles to financial outcomes, highlighting how lean tools drive both cost reduction and value improvement while sustaining an operational efficiency. Beyond savings, the system enhances staff experience and ensures timely consumable availability, supporting quality patient care.

Future study should explore long-term effects, adaptability in diverse settings and integration with digital tools. Broader implementation, continuous monitoring and combining Kanban with other lean strategies could amplify benefits and advance ‘*leanomics*’ for greater economic impact in healthcare supply management.

## Supplementary material

10.1136/bmjoq-2025-003416 online supplemental file 1

## Data Availability

Data are available upon reasonable request.
