# Supplementary material for: ‘Leanomics’ in healthcare: a three-year quality improvement study on the financial impact of a modified Kanban system in hospital storerooms
Source: BMJ Open Qual. 2025 Nov 12;14(4):e003416. doi: 10.1136/bmjoq-2025-003416 (PMC12612771; doi:10.1136/bmjoq-2025-003416)
Supplement: online supplemental file 1 [file bmjoq-14-4-s001.docx]

**Supplemental Materials**


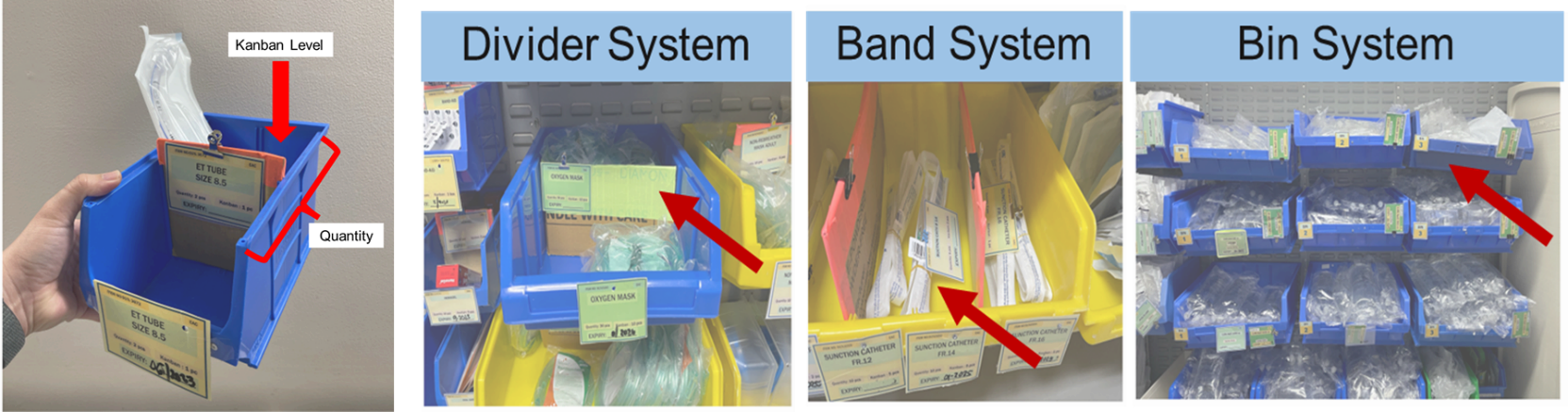
**Supplemental Figure 1. Total Quantity and Kanban Level in the Bin System and the Three Subsystems**


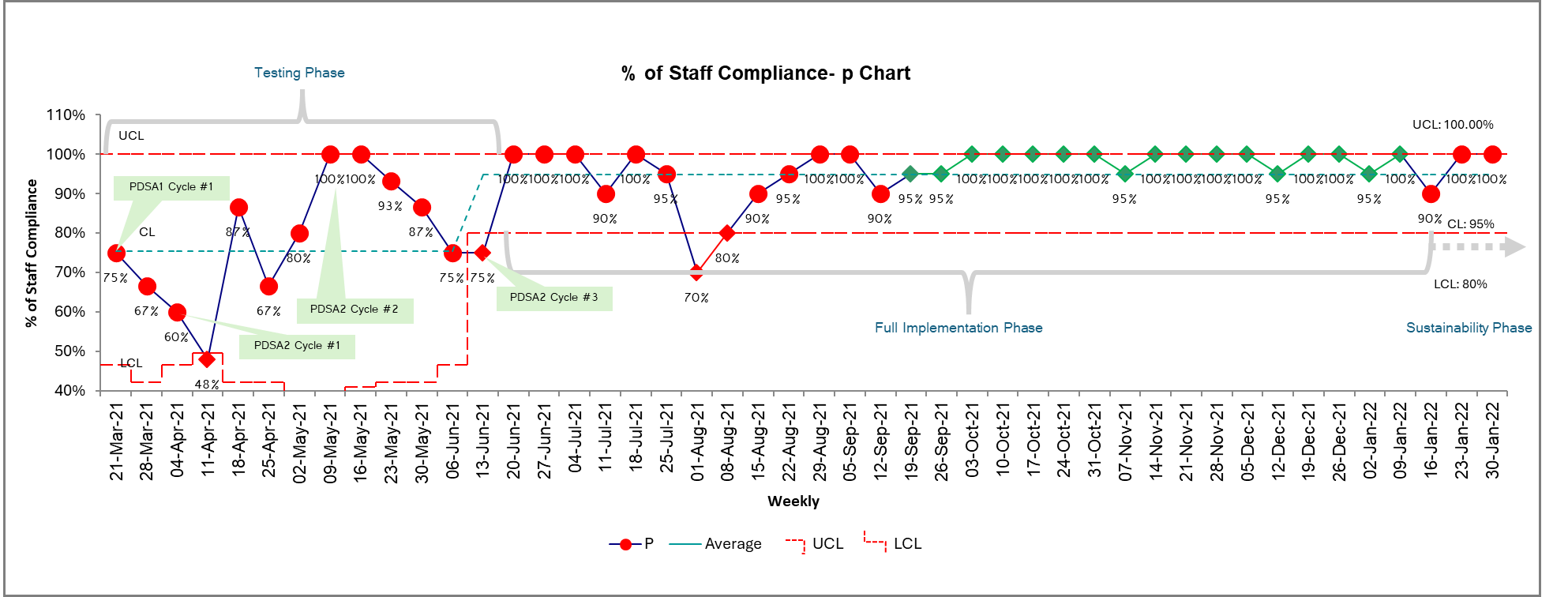
**Supplemental Figure 2. Staff Compliance p-chart**


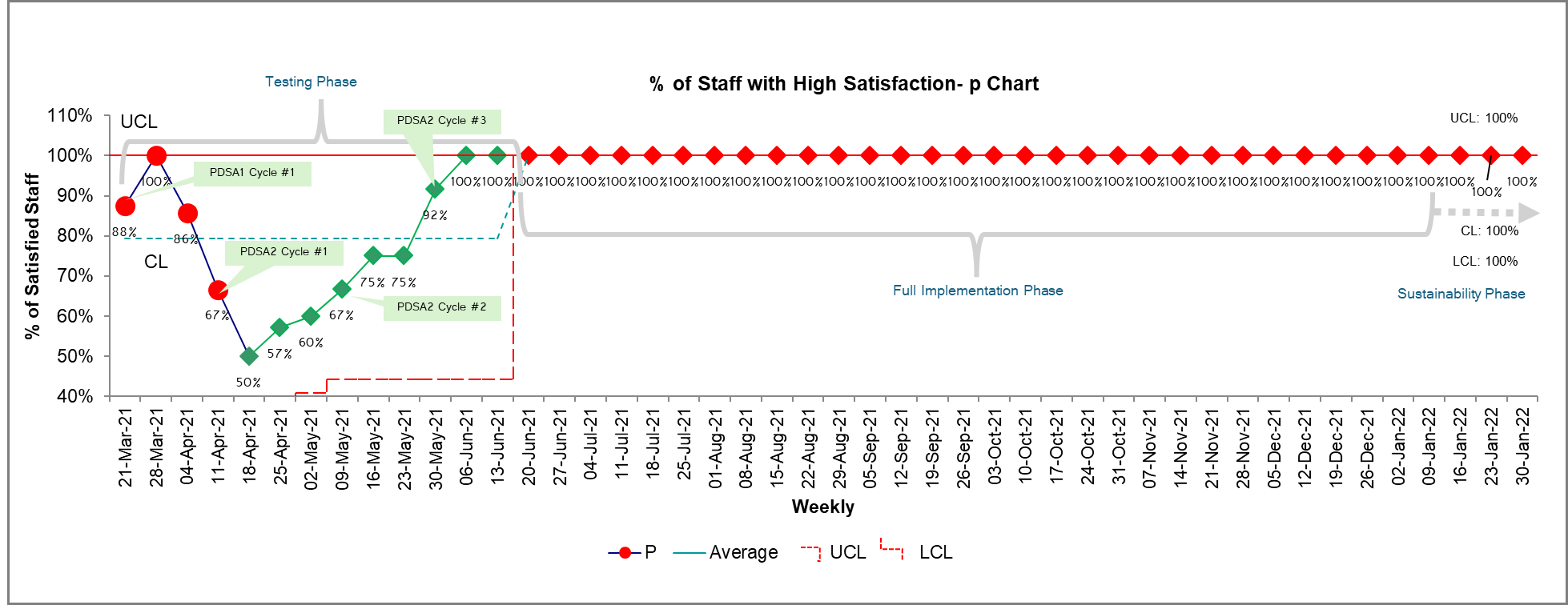
**Supplemental Figure 3. Staff Satisfaction p-chart**
